# Copper–zinc oxide heterojunction catalysts exhibiting enhanced photocatalytic activity prepared by a hybrid deposition method†

**DOI:** 10.1039/d1ra00691f

**Published:** 2021-03-10

**Authors:** José Montero, Tesfalem Welearegay, Jakob Thyr, Henry Stopfel, Tatjana Dedova, Ilona Oja Acik, Lars Österlund

**Affiliations:** Department of Materials Science and Engineering, The Ångström Laboratory, Uppsala University P. O. Box 35 SE-75103 Uppsala Sweden lars.osterlund@angstrom.uu.se; Department of Materials and Environmental Technology, Laboratory of Thin Film Chemical Technologies, Tallinn University of Technology Ehitajate tee 5 19086 Tallinn Estonia

## Abstract

Heterojunction copper–zinc oxide catalysts were prepared by a hybrid two-step methodology comprising hydrothermal growth of ZnO nanorods (ZnO-NR) followed by deposition of Cu_2_O nanoparticles using an advanced gas deposition technique (AGD). The obtained bicatalysts were characterized by SEM, AFM, XRD, XPS, PL and spectrophotometry and revealed well-dispersed and crystalline Cu_2_O nanoparticles attached to the ZnO-NR. The adsorption properties and photocatalytic degradation of Orange II dye in water solutions were measured. It was found that the bicatalysts exhibited a conversion rate and quantum yield that both were about 50% higher compared with ZnO-NR alone, which were attributed to the intrinsic electric field created at the p–n junction formed at the Cu_2_O/ZnO interface facilitating charge separation of electron–hole pairs formed upon interband photon absorption. The interpretation was evidenced by efficient quenching of characteristic deep level ZnO photoluminescence bands and photoelectron core-level energy shifts. By comparisons with known energy levels in Cu_2_O and ZnO, the effect was found to be most pronounced for the non-polar ZnO-NR side facets, which accounted for about 95% of the exposed surface area of the catalyst and hence the majority of dye adsorption. It was also found that the dye adsorption capacity of the ZnO nanorods increased considerably after Cu_2_O deposition thereby facilitating the oxidation of the dye. The results imply the possibility of judiciously aligning band edges on structurally controlled and well-connected low-dimensional semiconductor nanostructures using combined two-step synthesis techniques, where in particular vacuum-based techniques such as AGD allow for growth of well-connected nanocrystals with well developed heterojunction interfaces.

## Abstract 2

Hybrid synthesis of Cu_2_O/ZnO nanorod heterojunction exhibiting enhanced interfacial charge transfer and photocatalytic activity comprising hydrothermal synthesis step of ZnO nanorods followed by advanced gas deposition of Cu nanoparticles.

## Graphical abstract

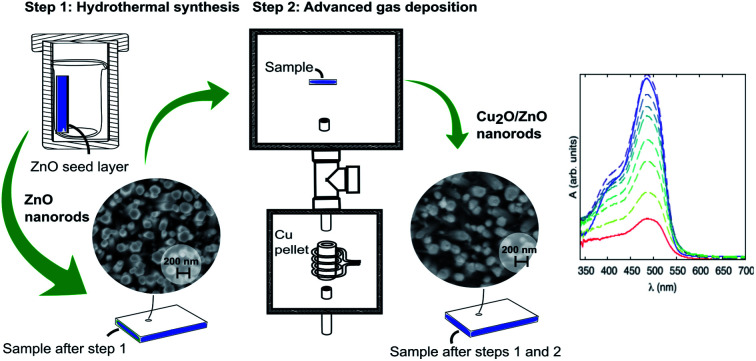

## Introduction

Ensuring adequate management of water resources is one of the UN's Sustainable Development Goals.^1^ However, despite considerable progress during the past decades,^2,3^ substances of anthropocentric origin continue to threaten freshwater reserves worldwide.^1,2^ Pollutants produced by human activity can be found not only in water reserves close to cities and towns, but also in remote and unexpected places.^4^ Human metabolites, pesticides, dyes, antibacterial agents, and pharmaceutical products to name a few, are complex chemical compounds that are not removed in wastewater treatment plants by conventional methods.^5^ Such persistent compounds reach lakes and rivers where they affect the flora and fauna and limit the availability of safe water for human and animal consumption.^2,3^ Advanced oxidation processes which involve the use of oxygen and hydroxyl radicals—such as photocatalytic degradation—have since 1970s evolved as promising methods for the removal of such persistent chemical compounds from wastewater, and can be operated under mild conditions meeting sustainable development goals.^5^

Zinc oxide (ZnO) has been shown to be a promising material for photocatalytic water cleaning.^6^ ZnO is easily fabricated by different techniques and generally exhibits good environmental stability.^6,7^ However, ZnO presents a disadvantage when the photocatalytic process is carried out under natural light illumination. The wide band gap of ZnO, about 3.3–3.4 eV,^7^ hinders the exploitation of other photons except of those in the deeper UVA region (*λ* < 375 nm). Furthermore, when ZnO nanoparticles are dispersed in waste water, they must be separated, and ideally recycled, before the cleaned water can be dispatched. In this sense, nanoporous coatings are advantageous for water cleaning. Synthesis of cuprous oxide (Cu_2_O) and cupric oxide (CuO) have been extensively studied, mostly using wet chemical reduction methods.^8,9^ Methods to prepare well-defined Cu_2_O nanoparticles by physical deposition methods remain, however, few, including *e.g.* laser ablation.^10^ Both CuO and Cu_2_O are p-type wide bandgap semiconductors. The photocatalytic and antibacterial properties of Cu_2_O and CuO have been recognized and methods to optimize the performance have been reported.^11–14^ It was reported the rate of photo-degradation of methyl orange is higher on that CuO nanowires than Cu_2_O nanowires, which was attributed to defects induced by the thermal treatment in vacuum to form Cu_2_O nanowires.^12^

In order to get the most out of the solar UV radiation, the quantum yield (*Φ*), *i.e.*, the number of degraded molecules per absorbed photon should be as high as practically possible. In addition, high product rate per mass of catalyst can be achieved using a nanostructured catalyst with a high surface-to-volume ratio. One common strategy involves the fabrication of nanostructured ZnO which addresses both of the above goals.^6^ To improve *Φ* for solar light illumination, it is essential to extend both the photon absorption into the visible part of the spectrum, but also to reduce electron–hole recombination and exploit all photo-excited electrons and holes efficiently. There are principally two ways to achieve that. First, low-dimensional materials, such nanorods, reduce the required diffusion length for the excited charge carriers. Second, if the lifetime of the excited electrons is prolonged, the probability that the electrons reach an active surface site is increased. A way to achieve this is to promote charge separation by, *e.g.* decorating the surface of ZnO, which present n-type character, with electrically well-connected nanoparticles of a p-type semiconductor with adequate band alignment, *i.e.*, by creating a p–n heterojunction,^15,16^ this is the so-called bicatalyst approach. Heterojunction Cu_2_O/ZnO catalysts have previously been prepared by combinations of magnetron sputtering, atomic layer deposition and electrodeposition,^17–19^ The importance of preparing stoichiometric Cu_2_O and ZnO interfaces to achieve interfaces that exhibit valance band offsets close to the thermodynamic limit (about 1.5 eV) and optimized photovoltaic properties have been emphasized. The position of band edges was reported to depend strongly on deposition conditions, and mis-aligned bands have been attributed to Fermi level pinning due to O vacancies in ZnO or Cu interstitials in Cu_2_O.^18^

In this paper, a hybrid method to fabricate bicatalysts consisting of ZnO nanorods decorated with Cu_2_O nanoparticles is presented. Our results show improved photodegradation of Orange II dye on these bicatalysts when compared with both uncoated ZnO nanorods and ZnO films. It is shown that the Cu_2_O/ZnO catalyst exhibits efficient charge transfer across the interface, and it is highlighted that the polar and non-polar ZnO nanorods facets have intrinsically different band edge energies that can be utilized to optimize redox reactions.

## Results and discussion

### Materials properties

**Fig. 1:**
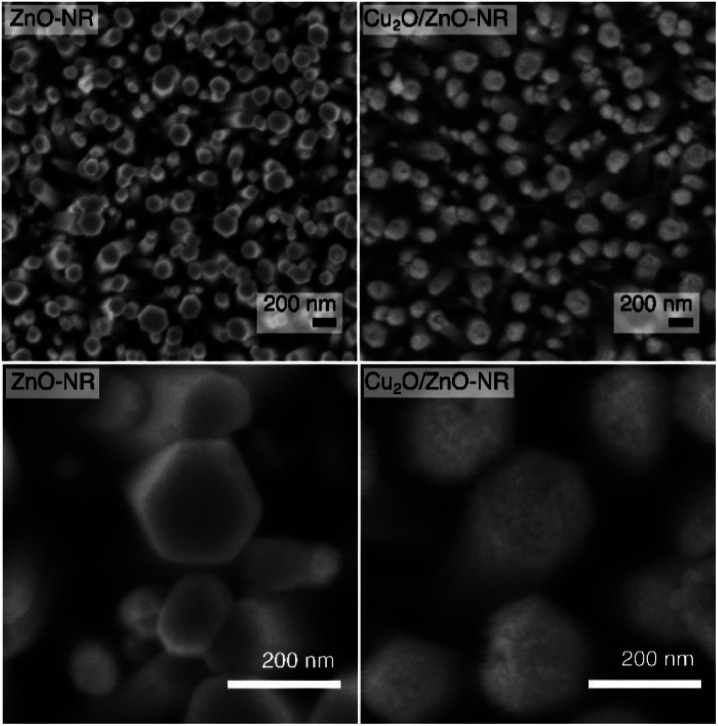
SEM images showing bare ZnO-NR (left panels) and Cu_2_O/ZnO-NR (right panels) with different magnifications (top and bottom panels) demonstrating the homogeneous Cu_2_O nanoparticle coverage on the ZnO-NRs.

Fig. 1 shows SEM images of Cu_2_O/ZnO-NR samples (right upper and lower panels) together with bare ZnO-NR samples prior to Cu nanoparticle deposition (left upper and lower panel). It is evident from Fig. 1 that the Cu_2_O particles cover not only the hexagonal (0001) top facets of the ZnO nanorods, but also the vertical {1-100}, {10-10} and {01-10} side facets. Analysis of the SEM images revealed that about 95% of the exposed surface area were side faces, and only about 5% are polar top faces—the average length of the hexagonal (0001) top facets is approx. 40 nm, while the average side length of the rods is approximately 300 nm. As we show below, this influences the relevant band alignments for the Cu_2_O–ZnO np-junctions that governs the observed physicochemical properties. AFM micrographs corresponding to ZnO-NR and Cu_2_O/ZnO-NR samples are presented in Fig. S1.† The average root mean square (RMS) roughness, determined by processing the AFM data with the software Gwyddion,^20^ was 75 nm and 66 nm for ZnO-NR and Cu_2_O/ZnO-NR, respectively. These values represent the topography of the nanorods rather than the surface roughness of the NRs themselves. The lower RMS for Cu_2_O/ZnO-NR is attributed to Cu_2_O particles covering holes, crevices and the space in-between the ZnO-NR (Fig. S1†). Additionally, a SEM micrograph corresponding to a Cu_2_O particulate film deposited onto a Si wafer is presented in Fig. S2.† Corresponding AFM line scans of a Cu_2_O particulate film are shown in Fig. S2.† It is seen that the surface roughness of the Cu_2_O particulate films deposited onto a Si wafer is smaller (RMS = 1.5 nm) than the one corresponding to the bare ZnO-f sample (RMS = 5.6 nm). The SEM images in Fig. 1 show however an apparent surface roughening on the scale of several tenth of nanometers, pointing to different scales of surface smoothening/roughening after Cu_2_O nanoparticle deposition on the ZnO-NR.

**Fig. 2:**
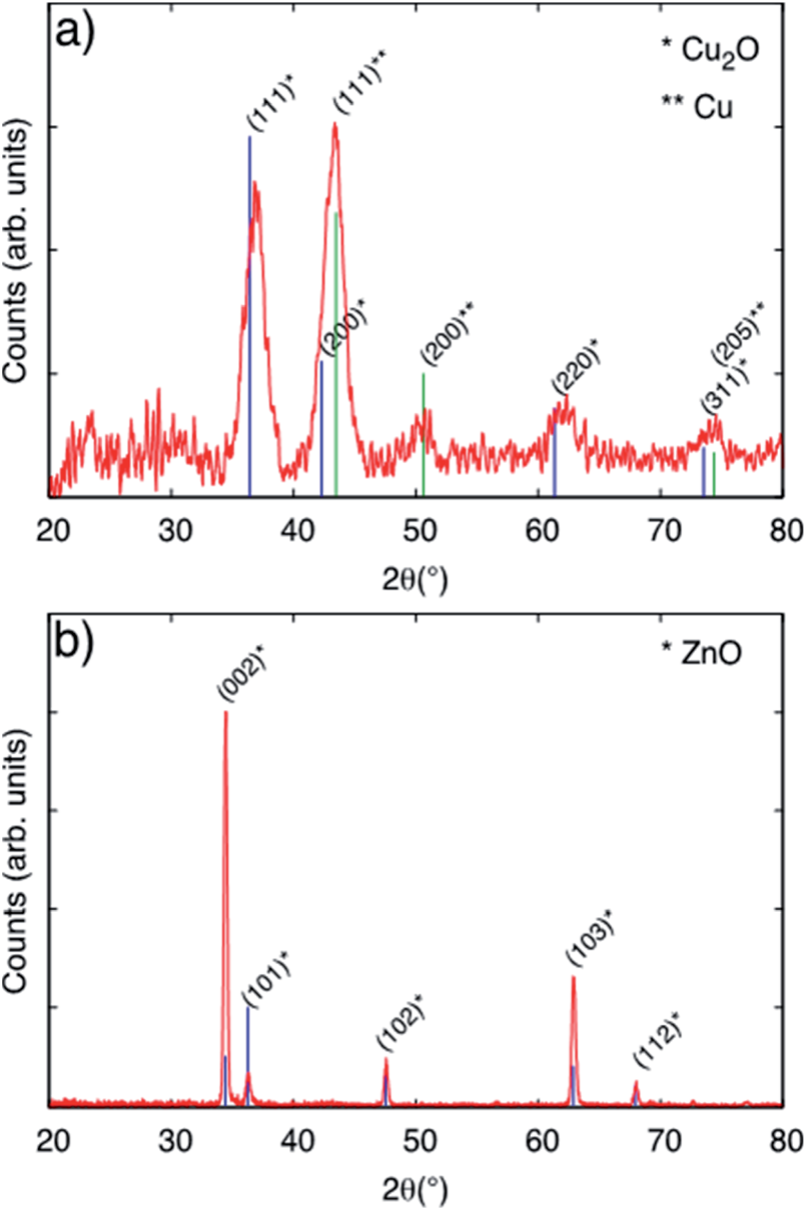
(a) GIXRD patterns corresponding to Cu_2_O nanoparticles deposited on flat glass substrate using equivalent coverage as the Cu_2_O/ZnO-NR samples. (b) Diffractogram corresponding to a Cu_2_O/ZnO-NR sample. Vertical lines correspond to standard diffraction reference cards (see text).

Fig. 2, panel (a) shows the diffractogram corresponding to a Cu-based particulate layer deposited on bare glass microscope slides. According to the diffractogram, the Cu_2_O nanoparticulate films exhibit peaks corresponding to metallic Cu and to the cubic Cu_2_O phase, which forms spontaneously by air exposure. The diffractograms in Fig. 2, panel (a) are assigned according to the Joint Committee of Powder diffraction Standards Cards JCPDS no. 04-009-2090 and 04-007-9767 for Cu and Cu_2_O, respectively. For Cu_2_O/ZnO-NR, the diffractogram is dominated by the features due to the hexagonal ZnO structure according to JCPDS no. 00-036-1451. The mean size of the crystalline Cu_2_O domains was calculated to be 5.3 nm by means of the Scherrer equation using the 〈111〉 reflection peak. Combining this information and the information obtained from Fig. S2,† it is inferred that the Cu_2_O particle size is the same as the crystalline domains size obtained from XRD. Fig. 2 panel (b) shows that the diffraction peaks corresponding to Cu or Cu oxides are masked by the much more intense ZnO peaks.

**Fig. 3:**
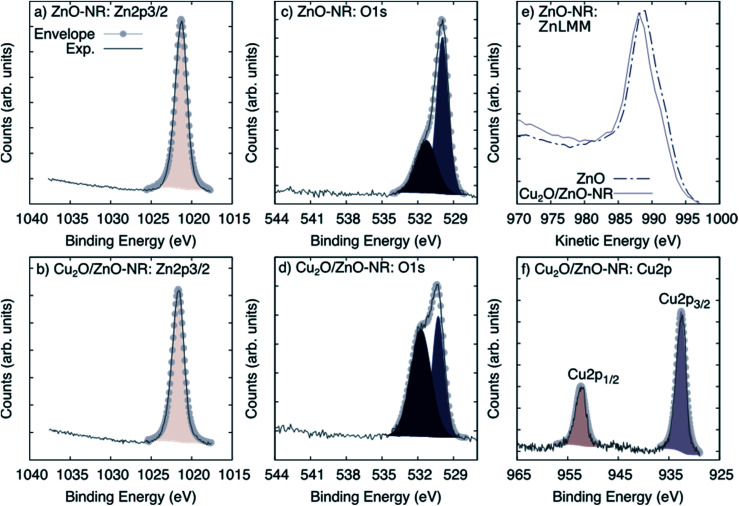
XPS spectra corresponding to (a and b) Zn 2p_3/2_, (c and d) O 1s, (e) the Auger transition Zn LMM for ZnO-NR and Cu_2_O/ZnO-NR and (f) the Cu 2p core-level spectra for Cu_2_O/ZnO.

Fig. 3 shows XPS spectra obtained on ZnO-NR and Cu_2_O/ZnO-NR in the binding energy regions corresponding to Zn 2p_3/2_ (panels (a) and (b)), O 1s (panels (c) and (d)), and the Auger transition Zn LMM (panel (e)). In addition, the Cu 2p transition is shown for the Cu_2_O/ZnO-NR sample. Shirley baseline correction was applied in each case. The signal corresponding to Zn 2p_3/2_ can be fitted to a single Gaussian–Lorentzian for all samples. The Zn 2p_3/2_ signal is located at 1021.3 eV for ZnO-NR (Fig. 3, panel (a)) and at 1021.6 eV for Cu_2_O/ZnO-NR (Fig. 3 panel (b)). In both cases, the component can be attributed to Zn–O bonds.^16,21^ The 0.3 eV energy difference points to charge transfer between the ZnO nanorods and the Cu_2_O nanoparticles.^16,21^ The blue-shift of the Zn 2p_3/2_ binding energies for the Cu_2_O/ZnO-NR is consistent with the concomitant decrease of the kinetic energy observed in the Zn LMM Auger transition (Fig. 3, panel (e)). The O 1s signal can for both the ZnO-NR and Cu_2_O/ZnO-NR be deconvoluted into two main components (Fig. 3, panels (c) and (d)). The component located at lower binding energy can be attributed to the O–metal bond,^21–23^*viz.* Zn–O and Cu–O in Cu_2_O/ZnO-NR (signal at 530.3 eV), and Zn–O in the ZnO-NR (signal at 530.9 eV). The other peak component in the O 1s region, located at 531.2 eV and 531.7 eV for bare and Cu_2_O coated ZnO-NRs, respectively, can be attributed to O–H,^24^ but also, in the case of Cu_2_O-coated samples, to Cu(OH)_2_.^25^ The latter assignment is consistent with the much higher relative intensity of the high-energy 531.7 eV O 1s peak (see Fig. 3, panel (d)). The XPS region corresponding to Cu 2p (Fig. 2, panel (f)) exhibits spin orbit splitting of Cu 2p_1/2_ and Cu 2p_3/2_. The relative intensity of these peaks is about 2 which is close to the theoretical multiplicity ratio (2J_3/2_ + 1)/(2J_1/2_ + 1).^23^ The binding energy shift is about 19.75 eV. Both Cu 2p spin orbit components can be deconvoluted into a single peak located at 932.5 (Cu 2p_3/2_) and 952.3 (Cu 2p_1/2_) eV. According to the XRD pattern shown in Fig. 2 panel (b), the nanoparticle layer is expected to be composed of a combination of Cu_2_O and Cu phases. The Cu and Cu_2_O contributions are however too close to be resolved by XPS [20]. In contrast, the absence of Cu^2+^ satellites that should be centered around 942 and 962 eV allows us to rule out the presence of CuO.^23^

**Fig. 4:**
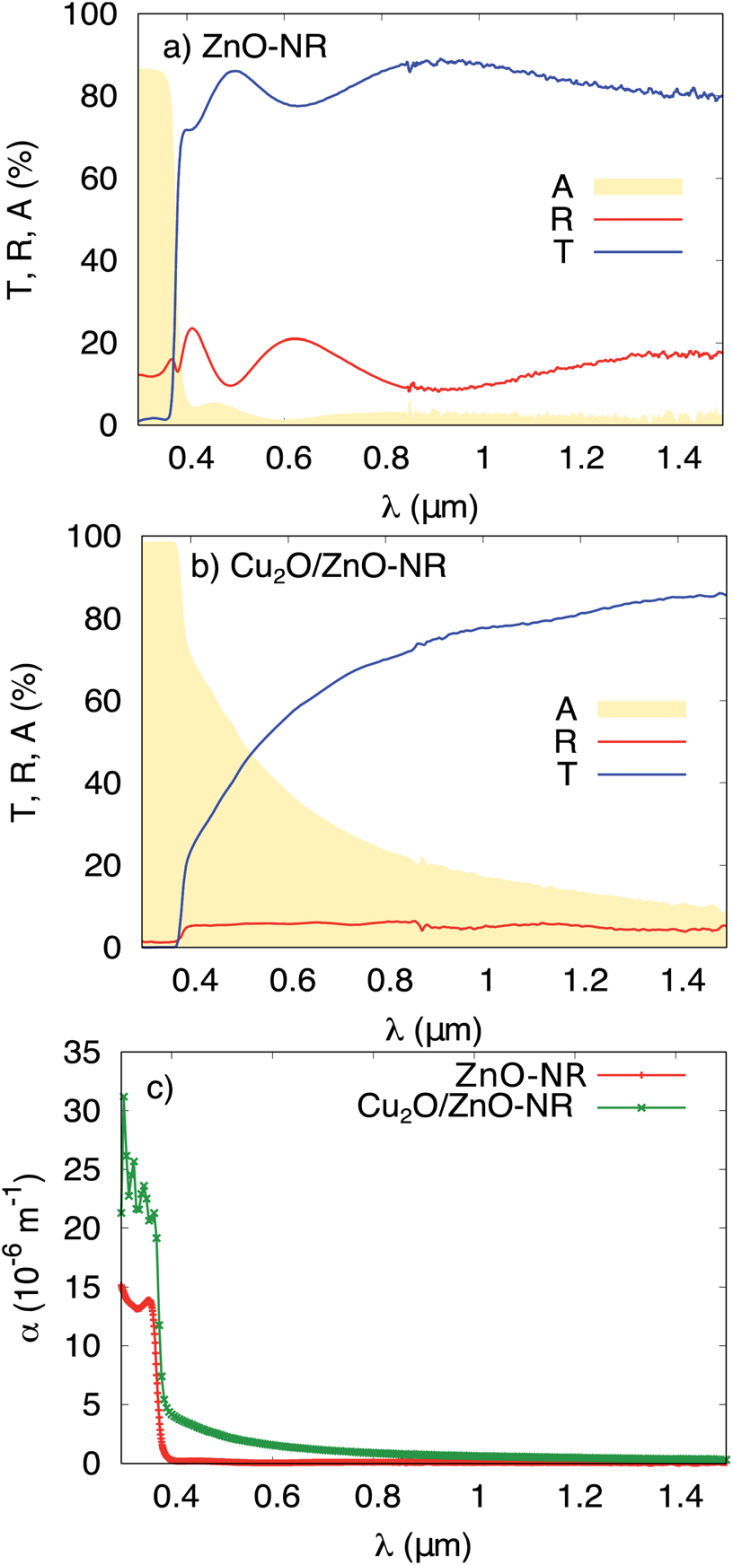
Transmittance *T*, reflectance *R* and absorbance *A* of (a) ZnO-NR deposited on glass, and (b) Cu_2_O/ZnO-NR. (c) The optical absorption coefficient *α* for ZnO-NR and Cu_2_O/ZnO-NR.

Fig. 4 shows the total transmittance *T*, reflectance *R*, and absorbance *A* (*i.e.*, *A* = 1 − *T* − *R*) corresponding to ZnO-NR and Cu_2_O/ZnO-NR, after the correction procedure by Roos.^26^ The absorption coefficient *α* was calculated using Hong's special absorption relation:^27^1
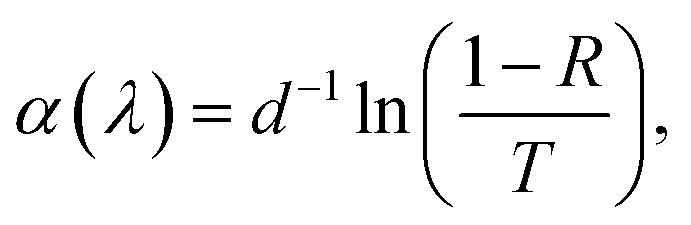
where *d* is the film thickness. Fig. 4 panel (c) shows *α* for both samples. The *T* and *R* spectra of the ZnO-NR samples are typical for a wide-band gap semiconductor, *i.e.*, high transparency in the visible and near infrared, where interference maxima and minima are observed (Fig. 4 panel (a)).^7^ Strong absorption is observed in the UV region due to the onset of interband absorption. ZnO is known to exhibit a direct optical band gap located between 3.3 and 3.4 eV,^7^ and consequently, *α* is expected to increase dramatically below 375 nm, which is seen in Fig. 4 panel (c). From the data presented in Fig. 4, using the usual Tauc-plot procedure, the band gap is estimated to be *E*_g_ = 3.3 eV for the direct allowed interband transition in the ZnO-NR samples. As discussed above, the as-deposited Cu_2_O nanoparticle film is mainly composed of Cu_2_O and metallic Cu nanoparticles. The presence of Cu_2_O, which exhibits a direct forbidden and direct allowed band gap transitions of 2.17 and 2.62 eV,^28^ respectively, results in a red-shift of the absorption edge in the Cu_2_O/ZnO samples compared with the ZnO-NR samples (Fig. 4 panel (c)). The presence of metallic Cu, even a thin film of few nanometers, would be expected to lead to a drastic increase of *R*, especially in the near-infrared region, which we can rule out based on our data.^29^ Instead, due to its particulate nature,^30^ the nanoparticle Cu_2_O layer on the ZnO-NRs causes an increase of *A* in the near infrared, as well as in the visible region (Fig. 4 panels (b) and (c)). We can thus infer that Cu_2_O/ZnO samples mainly consists of Cu_2_O particles.

### Adsorption and photocatalytic dye decomposition

The photocatalytic activity of the Cu_2_O/ZnO-NR, ZnO-NR and ZnO-f samples was assessed by means of photo-degradation of Orange II in aqueous solution. Before assessing the photocatalytic properties, however, several issues must be considered:^31^ The photocatalytic degradation must be distinguished from photolysis (degradation of the dye by the action of irradiation alone, *i.e.* without intervention of the catalyst) and from adsorption (dye molecules adsorbing on the catalyst surface, substrate and reactor walls without being decomposed). *In situ* optical transmission measurements of the solution under UV illumination were carried out without any sample submerged in the Orange II solution. Only a small decrease of the Orange II concentration was observed during the first hour of illumination, and subsequently the concentration remained stable during the following 8 hours of continuous illumination (Fig. S3†). Thus, we can rule out photolysis as a main pathway for Orange II removal.

**Fig. 5:**
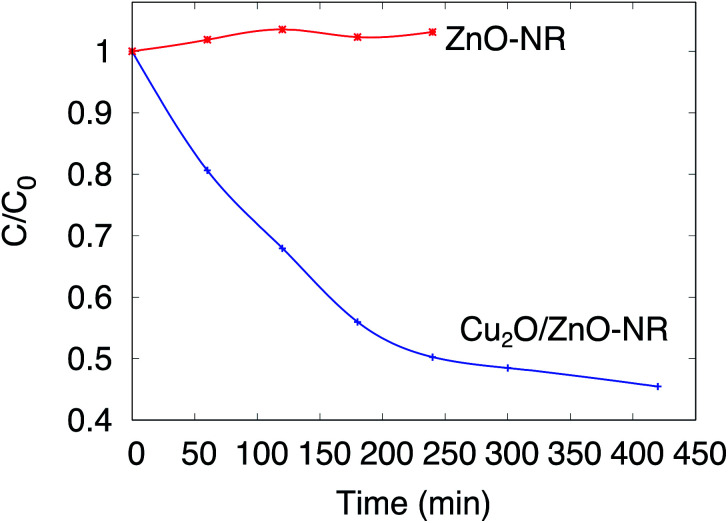
Normalized concentration of Orange II solution *C*/*C*_0_ as a function of sample immersion time. Measurements performed in darkness.

Fig. 5 shows *in situ* optical measurements with a sample immersed in the solution, but without illumination. In this figure, the normalized concentration *C*/*C*_0_ is plotted as a function of immersion time for ZnO-NR and Cu_2_O/ZnO-NR samples kept in darkness. The concentration of Orange II in the solution remains constant (or increases slightly due to evaporation) for uncoated ZnO-NR samples, but decreases abruptly on ZnO-NR samples coated with the Cu_2_O nanoparticles. Thus, the Cu_2_O nanoparticles that decorate the surfaces of the ZnO nanorods give rise to a dramatic increase of the adsorption capacity of the ZnO-NR sample. The results in Fig. 5 emphasizes the importance of distinguishing dye photodegradation from dye adsorption for Cu_2_O/ZnO-NR; it is evident that about 50% of the dye is adsorbed within 300 min, which for this particular adsorption system is very pronounced.

**Fig. 6:**
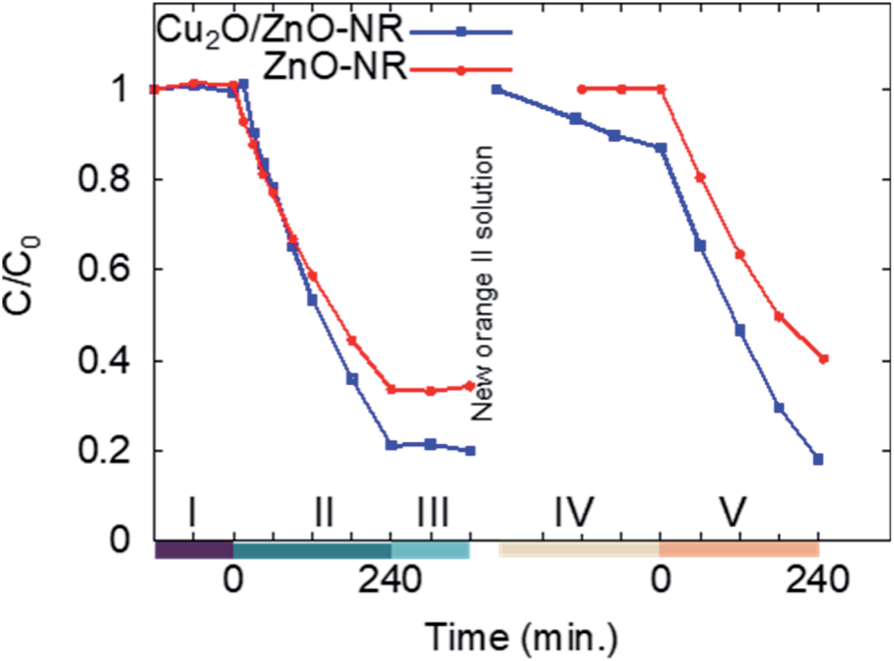
Time-evolution of the normalized concentration for ZnO-NRs and Cu_2_O/ZnO-NR, respectively, following 5 consecutive experiments performed in darkness (I, III and IV) and under illumination (II and IV). Between stages III and IV the Orange II solution was replenished.

Fig. 6 shows the result from two consecutive adsorption–photodegradation measurements, where a new fresh 50 μM Orange II solution was applied after the first illumination period. First, the samples were pre-soaked in Orange II solution and kept in darkness. This will, in principle, have no effect on the uncoated ZnO-NR samples. In contrast, for the Cu_2_O/ZnO-NR sample, the Cu_2_O surfaces will be saturated with Orange II molecules. Once the equilibrium is reached, the solution is replaced by a fresh solution, and once again the dye concentration is measured in darkness during 120 min, this stage is presented in Fig. 6 and labeled as I. As expected, the surface sites have been saturated and no further dye adsorption occurs for either ZnO-NR or Cu_2_O/ZnO-NR.

The samples are then illuminated with UV light (stage II). After 240 minutes illumination, significantly larger amounts of Orange II are removed from the solution with the Cu_2_O/ZnO-NR photocatalyst compared with ZnO-NR. After switching off the UV light, the residual concentration in the solution is monitored during 120 min in darkness (stage III). During stage III, a small decrease in *C* was observed for Cu_2_O/ZnO-NR which is attributed to Orange II adsorption. On the other hand, a small increase of *C* is observed for ZnO-NR, possibly due to water evaporation. In general, however, the flat curves in stage III, show that a stable remaining dye concentration is maintained in the solution, and that reversible photobleaching did not occur.^31^ After stage III, the solution is again replaced by 1 mL fresh 50 μM Orange II solution and the concentration monitored in darkness (stage IV). As expected, due to the photocatalytic degradation, the adsorption sites are replenished and again available for Orange II adsorption on the Cu_2_O/ZnO-NR samples. Thus, since Orange II saturation was not performed, dye adsorption on freed adsorption sites results in a reduced measured concentration in stage IV for Cu_2_O/ZnO-NR samples. In contrast, due to the negligible adsorption on ZnO-NR, no re-adsorption occurs on those samples, and *C* remains constant in stage IV for ZnO-NR. In stage V, UV light is again switched on, and again the Cu_2_O/ZnO-NR sample exhibits a higher photocatalytic degradation rate than ZnO-NR.

**Fig. 7:**
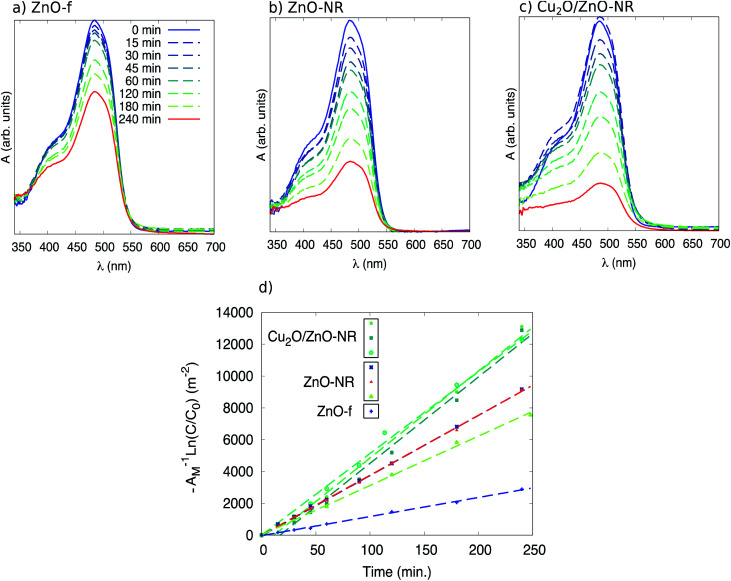
Optical absorbance spectra of Orange II solution as a function of sample illumination time in contact with (a) a ZnO-f, (b) ZnO-NR, and (c) Cu_2_O/ZnO-NR. In (d) a plot of −A^−1^ ln(*C*/*C*_0_) *vs.* illumination time is presented, where A_M_ is the macroscopic area of the films, *C*_0_ the initial concentration of Orange II in the solution and *C* the concentration at a given time.

Fig. 7 (panels (a), (b) and (c)) shows *in situ* absorbance spectra between 350 and 700 nm acquired during UV illumination during stage II in a ZnO film, ZnO-NR and Cu_2_O/ZnO-NR. It is evident that no clear absorbance due to photo-degradation byproducts is observed in the spectra, other than the main Orange II band at 484 nm and the shoulder at approximately 400 nm. It is, however, seen that during the first 15 minutes of illumination, the maximum in absorbance for Cu_2_O/ZnO-NR stays constant, or is even slightly increasing (Fig. 7, panel (c)), while the width of the peak increases. This suggests that during the first illumination period either (i) only pre-adsorbed Orange II molecules on the bicatalyst are photo-degraded, while the molecules in the solution remains intact (the *in situ* spectrophotometry only detects what is in the solution) and hence the absorption peak is roughly constant. Or, (ii) photo-desorption of Orange II occurs in the first 15 min from the “pool” of pre-adsorbed Orange II molecules into the solution and maintain a quasi-steady state concentration. We regard (i) as the most plausible explanation, which implies that it takes about 15 min under our experimental conditions to free adsorption sites to make them accessible for dye molecules in solution.

### Reaction kinetics

The evaluation of the photocatalytic degradation of Orange II was performed using the Langmuir–Hinshelwood model assuming first order kinetics.^32^ Thus, the pseudo-first order rate constant *k* can be related to the illumination time *t* through the expression2
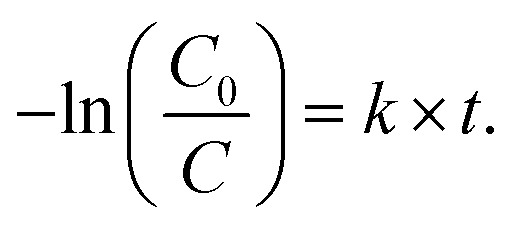


Plotting −ln(*C*_0_/*C*) as a function of *t* for the different sets of samples (ZnO-f, ZnO-NR and Cu_2_O/ZnO-NR) results in a collection of straight lines of slope *k*. Comparisons of the samples can be made after normalizing by the sample area. Accordingly, −ln(*C*_0_/*C*)/*A*_macro_ as a function of *t*, where *A*_macro_ is the macroscopic projected area of the sample, is presented in Fig. 7 panel (d).

Table 1 presents the relevant parameters extracted from the kinetic analysis. The data shown in Table 1 are average values obtained from the data plotted in Fig. 7. Table 1 shows the initial rate constant *k*(*t* = 0), as well as the initial rate constant normalized by the macroscopic (projected) area, *i.e. A*_macro_^−1^*k*(*t* = 0). Initial rate constants are however better compared using the microscopic area *A*_micro_. Analysis of several 2 × 2 μm^2^-sized AFM images revealed that *A*_micro_ is 1.8, 1.9 and 1.4 times *A*_macro_ for ZnO-NR, Cu_2_O/ZnO-NR and ZnO-f, respectively (see ESI†). The corresponding reaction rates *A*_micro_^−1^*k*(*t* = 0) are also presented in Table 1.

**Table 1:** Parameters from pseudo-first order kinetic analysis of Orange II dye photo-degradation. *k*(*t* = 0) is the initial pseudo-first order rate constant, FQE is the formal quantum efficiency, *Φ* is the quantum yield, and *A*_macro_ and *A*_micro_ are the macroscopic and microscopic sample areas, respectively (see text)

Sample	*k*(*t* = 0), mol min^−1^ (×10^13^)	*A*_macro_^−1^ *k*(*t* = 0), μmol (m^2^ min)^−1^	*A*_micro_^−1^ *k*(*t* = 0), μmol (m^2^ min)^−1^	FQE, mol per incident photon (×10^−5^)	*Φ*, mol per absorbed photon (×10^−5^)
ZnO-f	5.18	0.60	0.43	1.52	1.57
ZnO-NR	14.4	1.81	1.00	4.42	4.55
Cu_2_O/ZnO-NR	23.5	2.71	1.43	6.91	6.91

The formal quantum efficiency FQE and quantum yield (*Φ*) are also presented in Table 1. FQE refers to the number of degraded molecules per incident photon, while *Φ* is the number of degraded molecules per absorbed photon.^33^ For this purpose, the source has been considered as ideally monochromatic (365 nm) and able to provide a photon flux of 
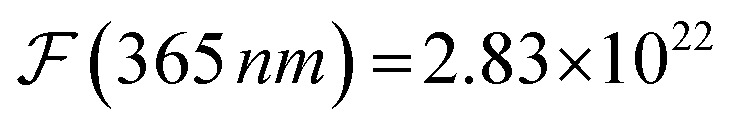
 photons min^−1^ m^−2^. The absorbed photons by the sample can be calculated by the expression:3
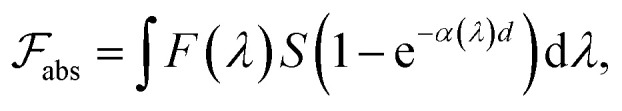
where *S* is the illuminated cross-section of the film. From the data in Fig. 4 we have *α*(365 nm) = 1.0 × 10^7^ m^−1^ for ZnO-f and ZnO-NR, and 2.1 × 10^7^ m^−1^ for Cu_2_O/ZnO-NR samples. Therefore, practically all photons that impinge on the surface on any of the samples will be absorbed. As mentioned in the experimental section, due to size limitations of the experimental set-up, the light beam impinges at an angle of 45° with respect to the sample surface normal, thus *S* = 1.4 cm^2^ (hence *S* is not the same as *A*_M_). For the calculation of FQE and *Φ*, all the photons in *S* are considered to impinge onto an active (coated) region of the sample.

The Orange II dye removal rate, FQE and *Φ* are unequivocally largest on the Cu_2_O/ZnO-NR sample compared with both ZnO-NR and ZnO-f. The lowest rate and efficiency are found for ZnO-f. In particular, the macroscopic rate is about 50% larger and *Φ* is 52% larger, respectively, for Cu_2_O nanoparticle coated ZnO-NR compared to a similar uncoated ZnO-NR samples, and both values are about 4.5 times larger than the thin ZnO film. We note that the values given in Table 1 for the Cu_2_O/ZnO-NR case can be considered as a lower limit, and that the actual rate and efficiency corresponding to Cu_2_O/ZnO-NR are certainly larger than those presented. This is due to two main factors: (i) during the AGD deposition of the Cu_2_O particles, the sample holder prevents deposition onto the borders of the sample. Therefore, the ZnO-NR area that is coated with Cu_2_O particles is slightly smaller than *A*_M_. Thus, the parameters for Cu_2_O/ZnO-NR presented in Table use an overestimated surface area. (ii) In addition, the Cu_2_O/ZnO-NR samples were exposed to an extra amount of pre-adsorbed dye molecules to compensate the reduced dye concentration in solution due to dark adsorption (see Fig. 6, stage I), which is neglected when calculating the parameters in Table 1. Nevertheless, despite this conservative estimate, the improvement achieved by the Cu_2_O/ZnO-NR bicatalyst is evident.

**Fig. 8:**
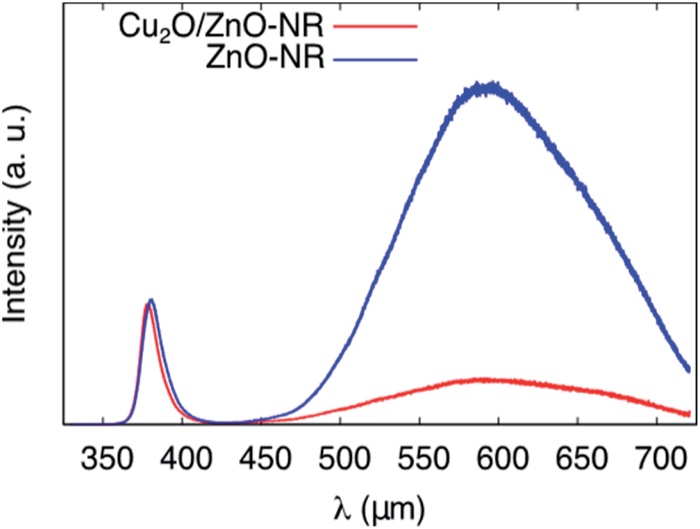
Photoluminescence spectra of ZnO-NR and Cu_2_O/ZnO-NR obtained at *T* = 295 K showing the interband D^0^X and deep level (DL) emission peaks.

To gain further insights into the synergetic effect of the Cu_2_O/ZnO-NR bicatalyst, PL measurements were performed at room temperature for both ZnO-NR and Cu_2_O/ZnO-NR. Representative spectra are shown in Fig. 8. The PL emission is clearly dominated by the ZnO contribution, which consists of a near band emission (NBE) located at 380 nm (3.26 eV) and a broader deep level emission located in the visible region, between 450 and 700 nm (2.7 and 1.7 eV).^34^ After coating the ZnO-NR with Cu_2_O, the deep level emission (DL) is strongly quenched. This deep level emission is usually attributed to surface defect states. On the other hand, the near band emission remains almost unaltered. Analogous to the XPS results, indicating a charge transfer from ZnO to Cu_2_O, the PL results clearly show that the ZnO surface states are depleted upon contacting the NRs with Cu_2_O nanoparticles.

Table 2 shows that quenching of the deep level emission (expressed by the DL/D^0^X intensity ratio) is much less pronounced using wet-chemically prepared ZnO nanoparticles deposited on Cu_2_O films prepared by magnetron sputtering as described elsewhere,^13^ emphasizing the importance of physically well-connected Cu_2_O and ZnO interfaces for efficient interfacial charge transfer.

**Table 2:** Intensity ratio DL/D^0^X of the measured photoluminescent transition for ZnO nanorods (ZnO-NR), Cu_2_O nanoparticle coated ZnO-NR (Cu_2_O-NP/ZnO-NR), ZnO nanoparticles (ZnO-NP) and ZnO nanoparticles on nanoporous Cu_2_O films (ZnO-NP/Cu_2_O-film) and the corresponding standard deviations and peak positions *λ*

Sample	DL/D^0^X intensity ratio	Std. dev, *σ*	DL, *λ* (nm)	D^0^X, *λ* (nm)
ZnO-NR	2.63	0.11	595	381
Cu_2_O-NP/ZnO-NR	0.42	0.08	595	378
ZnO-NP	3.76	0.28	583	368
ZnO-NP/Cu_2_O-film	3.74	0.26	583	364

**Fig. 9:**
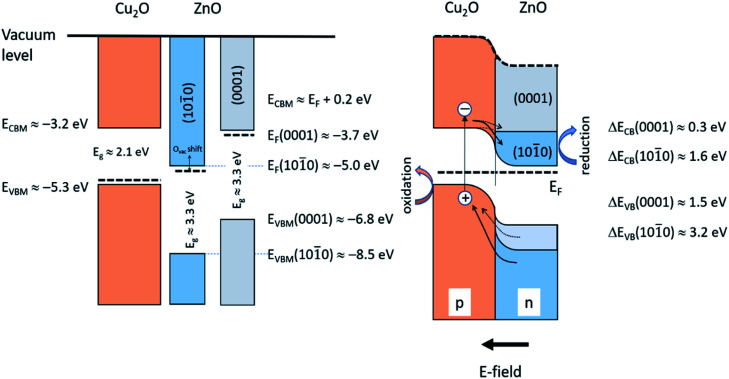
Schematic illustration of the isolated Cu_2_O and ZnO-NR (left) and the Cu_2_O/ZnO-NR heterojunction (right). Data adapted from ref. 32–36, and this work.

Fig. 9 illustrates schematically the band energy diagrams of the valence band (VB) and conduction band (CB) regions for the isolated and electronically connected Cu_2_O and ZnO-NR systems, respectively. Interband transition in Cu_2_O close to the space charge region leads to charge separation by diffusion of electrons to CB of ZnO, while interband transition in ZnO results in hole diffusion to the VB of Cu_2_O. The hole oxidation occurs on the Cu_2_O, while the electron reduction occurs on ZnO. Both transitions are accessible with the 365 nm photons employed. If appreciable surface states of ZnO-NR are included, the Fermi level is pinned,^35^ which reduces the charge separation efficiency of the device. Thus, a balance between nanostructure and crystallinity is expected to optimize the charge separation of the heterojunction device. Our optical data show no pronounced bandgap widening due to a Burstein–Moss effect, thus suggesting small or moderate influence of O vacancy defects. An important difference of the p–n junction properties of Cu_2_O connected to the non-polar and polar surfaces of the ZnO-NR, respectively, is that the work function of the non-polar ZnO is much larger than the polar (0001) surface. Using relevant reported data on the work function, band edge positions of Cu_2_O and the ZnO surfaces,^18,19,36–38^ it is seen in Fig. 9 that the electric field formed between the Cu_2_O and non-polar ZnO side facets is much larger than on the ZnO (0001) surface. We can thus anticipate a much more efficient charge separation on the side facets of the ZnO-NRs. Since the majority of exposed samples surface constitute non-polar ZnO side facets, we conclude that the main effect we observe is due to reactions on Cu_2_O attached on the ZnO side facets. In Fig. 9 the role of the heterojunction in the photo-degradation reaction is also illustrated. Interband transition due to light absorption near the junction, or electron–hole pairs that reach the junction become separated, whereby electrons are injected from the CB of Cu_2_O to the CB of ZnO-NR, while holes are injected from the VB of ZnO-NR to the VB of Cu_2_O. Thus, Cu_2_O would work as the oxidation catalyst and ZnO-NR as the reduction catalysts. The strong adsorption of Orange II on the Cu_2_O/ZnO-NR in comparison with ZnO-NR (see Fig. 6) shows that Orange II adsorb on Cu_2_O and facilitates the oxidation reaction. Note that upon illumination with light >375 nm, only Cu_2_O is photo-active, but still would work as a biocatalyst. However, due light absorption in the Orange II dye, only light that access the transparent dye “window” in the UVA that promote interband transitions in both oxides are employed in this study.

## Experimental

### Materials synthesis

**Fig. 10:**
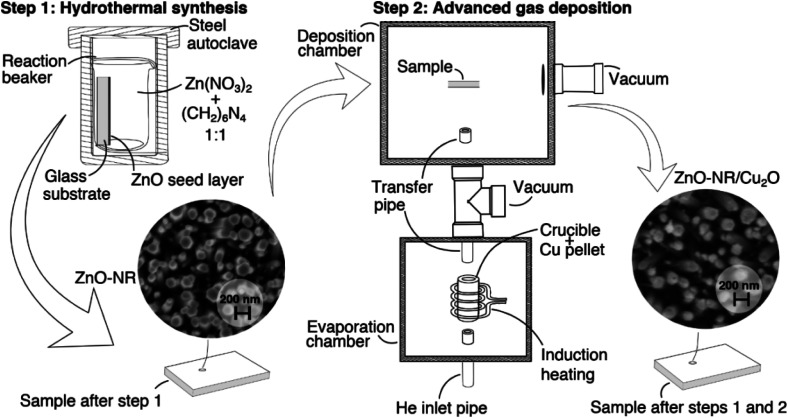
Schematic flow chart depicting the hybrid synthesis methodology for preparing Cu_2_O/ZnO-NR samples comprising a hydrothermal synthesis step to make ZnO nanorods (NR) followed by advanced gas deposition of size-controlled Cu nanoparticles in a two-stage vacuum deposition chamber.

Fig. 10 shows a schematic flow chart of the hybrid synthesis methodlogy used for preparing Cu_2_O/ZnO-NR samples. Crystallized ZnO nanorods (ZnO-NR) fixed onto a glass substrate were used as a scaffolding structure for deposition of copper oxide nanoparticles. ZnO-NR uniform layers were grown by hydrothermal synthesis method as described elsewhere.^39^ Briefly, glass substrates covered with ZnO seed coatings were placed in 40 mL of a reactor container with a mixture of 0.1 mol L^−1^ zinc nitrate hexahydrate (Zn(NO_3_)_2_·6H_2_O, Sigma-Aldrich, 99.9%) and hexamethylenetetramine ((CH_2_)_6_N_4_, HMT) 0.1 mol L^−1^ aqueous solutions. The reactor container was then transferred to a steel autoclave and the reaction proceeded for 2 hours at 120 °C. For comparison, ZnO films (ZnO-f) were deposited by pneumatic spray pyrolysis at substrate temperature 450 °C using 0.1 mol L^−1^ zinc acetate solution in isopropyl alcohol (2/3 vol.).^40^

Copper oxide (Cu_2_O) nanoparticles were deposited onto the ZnO-NR samples (denoted Cu_2_O/ZnO-NR) by an inert gas deposition technique, commonly denoted advanced gas deposition (AGD),^41^ which produces ultra-pure particles with narrow (log-normal) size distribution under vacuum deposition conditions. We emphasize that purity, offered by the vacuum deposition, is crucial to obtain nanoparticles with good physical contact to the ZnO-NRs. The AGD set-up consists of two separated vacuum chambers, a deposition chamber and an evaporation chamber, which are maintained at different pressures and connected by a transfer pipe (3 mm diameter). The pressure in the evaporation chamber is higher than at the deposition chamber, thus, a gas flow is stablished between them.^41^ A schematic representation of the AGD set-up used for the deposition of the Cu_2_O particles can be found in our previous study.^42^ In the evaporation chamber, Cu is evaporated from a metallic Cu pellet (purity 99.95%) by induction heating in an inert atmosphere (in this case He). The saturated metal vapor condenses in the inert He flow in the melt zone above the heated Cu pellet, forming a log-normal size distribution of Cu metal nanoparticles that can be controlled by the metal vapor pressure (or the Cu target temperature). The nanoparticles are then transported in the He gas through the transfer pipe to the deposition chamber, which is held at lower pressure, where they impact on the ZnO-NR sample substrates. The substrate is attached to a motorized XYZ sample holder, and a pre-programmed deposition pattern is employed to achieve homogeneous deposition on the sample. The parameters that can be controlled are the induction coil power *P*_i_ (target temperature), pressure difference between the chambers (Δ*P*), He flow (*ϕ*_He_) and XYZ sample holder path and speed. In this work, a set of Cu_2_O nanoparticulate samples was prepared using Δ*P* = 160 mbar, *ϕ*_He_ = 20 L min^−1^ and *P*_i_ = 3.5 kW. The substrate holder speed was fixed to 2.5 mm s^−1^. No reactive gas (O_2_) were used during Cu nanoparticle deposition. Air exposure at room temperature yields primarily Cu_2_O nanoparticles (with a minor fraction of metal Cu, that presumably is located in the particle's core), which for simplicity here we denote Cu_2_O. This composition is stable throughout the measurements and sample handling. Further oxidation yield transformation to CuO, in agreement with previous studies.^8,12^ At elevated temperatures it is possible to successively obtain CuO, Cu_3_O_4_, and eventually CuO_2_.^13^ The Cu particle deposition rate was calibrated by independent deposition of Cu nanoparticles on a flat glass substrate and the resulting film thickness was determined by profilometry (Bruker DektakXT). In this way, the equivalent thickness of the Cu_2_O layer that was deposited on the ZnO-NRs was estimated to be 36.7 ± 1.3 nm.

### Materials characterization

The surface morphology of the different samples where measured by scanning electron microscopy (SEM) using a LEO 1550 instrument. Surface roughness was measured by atomic force microscopy (AFM) employing a Bruker Dimension Icon AFM in ScanAsyst mode. The crystalline structure was studied by grazing incidence X-ray diffraction (GIXRD) using a Siemens D5000 instrument at 1° angle of incidence. The surface oxidation state of the different chemical elements was investigated by X-ray photoelectron spectroscopy (XPS) using an Ulvac PHI Quantera II instrument. The binding energy was calibrated from the C–C contribution due to the C 1s adventitious carbon signal at 284.8 eV. The optical transmittance *T*, reflectance *R* and absorbance were measured in a Perkin Elmer Lambda 900 spectrophotometer equipped with an integrating sphere. Photoluminescence (PL) measurements were performed with a Renishaw Invia Reflex micro-Raman system equipped with 40× NUV objective and using a 325 nm laser excitation source and a 2400 lines per mm grating. The spectral calibration was performed with a diamond reference sample with a signal peak at 1332 cm^−1^ Raman shift. The samples were measured with an acquisition time of 10 s. The laser power at the sample was approximately 0.1 mW, which did not result in transformation of Cu_2_O into other oxide phases.

### Photocatalytic experiments

Orange II (C_16_H_11_N_2_NaO_4_S, CAS number 633-96-5, Sigma-Aldrich) was used as a test molecule for dye absorption and photocatalytic experiments. For this purpose, a 50 μM Orange II solution in de-ionized water was prepared.

ZnO-f, ZnO-NR and Cu_2_O/ZnO-NR samples with a projected geometric area of approximately 1.4 × 1.4 cm^2^ were submerged in 1 mL of Orange II solution in a quartz cuvette (transparent in the UVA and visible region) and illuminated with a UVA LED light source (Thorlabs LED diode 365 nm, FWHM = 7.5 nm, 25.7 mW cm^−2^) with an incident angle of approximately 45° to allow for simultaneous spectrophotometric measurements. The excitation wavelength was chosen to be outside the absorption range of Orange II, *i.e.*, in the UV-A region, outside of the visible region, and energetic enough for exciting electrons across the bandgap in both ZnO and Cu_2_O. The latter will facilitate the comparison between ZnO-NR and Cu_2_O/ZnO-NR samples, while the former prevents absorption of the excitation light by the dye.

Changes in the dye Orange II concentration, *C*, in the solution were monitored by the usual photochromatic method,^31^ establishing a direct relationship between initial concentration *C*_0_ = 50 μM, and the initial maximum in the optical absorbance, located at 484 nm. The absorbance of the solution Orange II solution was measured *in situ*, between 350 and 800 nm, using an Ocean optics HR4000CG-UV-NIR spectrophotometer.

## Conclusions

Heterojunction bicatalysts based on nanostructures of ZnO and Cu_2_O were prepared by a two-step fabrication method. First, ZnO nanorods with good crystallinity, were prepared by hydrothermal growth onto glass substrates. These ZnO nanorods were used as scaffolding for the deposition of Cu_2_O nanoparticles by a vacuum based gas deposition method. The resulting Cu_2_O/ZnO-NR bicatalysts showed about 50% improvement of both the overall reaction rate and the quantum yield (moderate estimation) compared with the bare ZnO-NRs for the photocatalytic degradation of Orange II in aqueous solutions. This effect is attributed to improved charge separation of the photo-excited electrons and hole pairs at the Cu_2_O/ZnO heterojunction as evidenced by both XPS and PL data. Good crystallinity and electrically well-connected Cu_2_O–ZnO interfaces are crucial to obtain efficient charge transfer. The larger work function of the non-polar ZnO side facets compared with the top polar (0001) facet implicate facet dependent reactivity, and a much larger internal electrical field created in the Cu_2_O/ZnO junctions on the non-polar side facets. Defect states in ZnO is expected to deteriorate the activity, especially on the (0001) surface. Besides the photocatalytic activity, a large increase of the adsorption of Orange II molecules is observed for the Cu_2_O/ZnO bicatalyst when compared with uncoated ZnO-NR.

## Author contributions

J. Montero: investigation, formal analysis, writing – original draft preparation. T. Welearegay: investigation. J. Thyr: investigation. H. Stopfl: investigation. T. Dedova: investigation. I. Oja Acik: conceptualization, Methology. L. Österlund: conceptualization, methodology, writing – reviewing and editing, supervision.

## Conflicts of interest

There are no conflicts to declare.

## Supplementary Material

RA-011-D1RA00691F-s001
