# Correction: Copper–zinc oxide heterojunction catalysts exhibiting enhanced photocatalytic activity prepared by a hybrid deposition method

**DOI:** 10.1039/d1ra90096j

**Published:** 2021-04-13

**Authors:** José Montero, Tesfalem Welearegay, Jakob Thyr, Henry Stopfel, Tatjana Dedova, Ilona Oja Acik, Lars Österlund

**Affiliations:** Department of Materials Science and Engineering, The Ångström Laboratory, Uppsala University P. O. Box 35 SE-75103 Uppsala Sweden lars.osterlund@angstrom.uu.se; Department of Materials and Environmental Technology, Laboratory of Thin Film Chemical Technologies, Tallinn University of Technology Ehitajate tee 5 19086 Tallinn Estonia

## Abstract

Correction for ‘Copper–zinc oxide heterojunction catalysts exhibiting enhanced photocatalytic activity prepared by a hybrid deposition method’ by José Montero *et al.*, *RSC Adv.*, 2021, **11**, 10224–10234, DOI: 10.1039/D1RA00691F.

The authors regret that the funding information was incorrectly shown in the acknowledgements section of the original manuscript. The corrected funding acknowledgement is as shown below.

This work was supported by Swedish research counsel VR (grant 2016-05904), Swedish research counsel FORMAS (grant 2016-00908), the Estonian Research Council grant PRG627, and the Estonian Centre of Excellence project TK141.

The Royal Society of Chemistry apologises for these errors and any consequent inconvenience to authors and readers.

## Supplementary Material

