# Supplementary material for: Copper–zinc oxide heterojunction catalysts exhibiting enhanced photocatalytic activity prepared by a hybrid deposition method
Source: RSC Adv. 2021 Mar 10;11(17):10224–34. doi: 10.1039/d1ra00691f (PMC8695732; doi:10.1039/d1ra00691f)
Supplement: RA-011-D1RA00691F-s001 [file RA-011-D1RA00691F-s001.pdf]

Electronic Supplementary Material (ESI) for RSC Advances.
This journal is © The Royal Society of Chemistry 2021

Supplementary Information: Copper-Zinc Oxide Heterojunction Catalysts Exhibiting Enhanced Photocatalytic Activity Prepared by A Hybrid Deposition Method

José Montero,[a] Tesfalem Welearegay,[a] Jakob Thyr,[a] Henry Stopfel,[a] Tatjana Dedova,[b] Ilona Oja Acik,[b] and Lars Österlund*[a]

Figure S1 shows AFM micrographs corresponding to ZnO-NR and ZnO-NR/Cu_2O films. The root-mean-square (RMS) roughness determined from these measurements is 75.0 nm and 66.0 nm for ZnO-NR and Cu_2O/ZnO-NR, respectively. Figure S2 shows an SEM image and correspinding AFM line scans of Cu_2O on a silicon wafer.

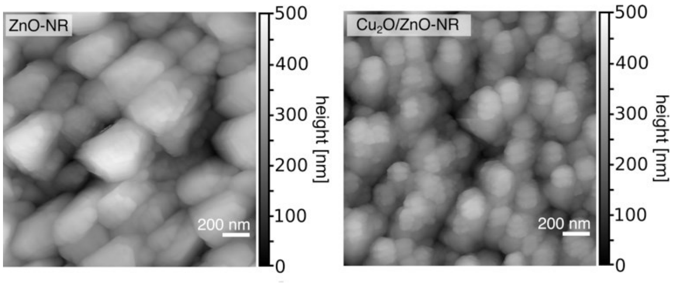

Figure S1 AFM images showing bare ZnO-NR (left panel) and Cu_2O/ZnO-NR (right panel).

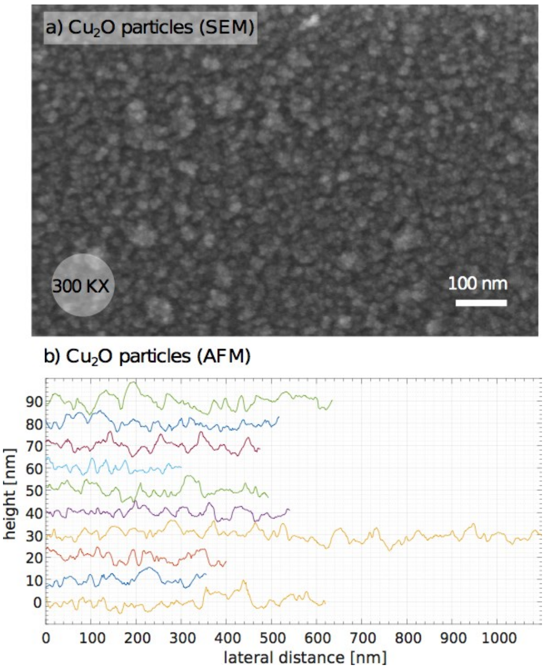

Figure S2 (a) SEM micrograph corresponding to the Cu_2O particles deposited onto a Si-wafer substrate. (b) AFM line scans extracted from AFM images. The curves are offset along the ordinate axis by 10 nm for clarity. On average, the RMS roughness was 1.5 nm.

a. Department of Materials Science and Engineering, The Ångström Laboratory, Uppsala University, P.O. Box 35, SE-75103 Uppsala, Sweden.
b. Department of Materials and Environmental Technology, Laboratory of Thin Film Chemical Technologies, Tallinn University of Technology, Ehitajate tee 5, 19086 Tallinn, Estonia.
* Corresponding author email address: lars.osterlund@angstrom.uu.se
Electronic Supplementary Information (ESI) available: [details of any supplementary information available should be included here]. See DOI: 10.1039/x0xx00000x

Figure S3 shows the normalized concentration of Orange II in solution as a function of illumination time without any sample immersed in the solution. Except for a small drop taking place during the first 60 min, the concentration of Orange II remains unaltered even after 520 min of constant illumination, ruling out photolysis as the main degradation path. The initial drop, which is much smaller than the photo-degradation in presence of the catalysts, and highly reproducible can be attributed to light-induced adsorption of Orange II molecules to the walls of the cuvette. This small effect, which will contribute with a small offset in our photocatalytic experiments, has been omitted in the quantitative analysis of the photo-degradation rate presented in the paper.

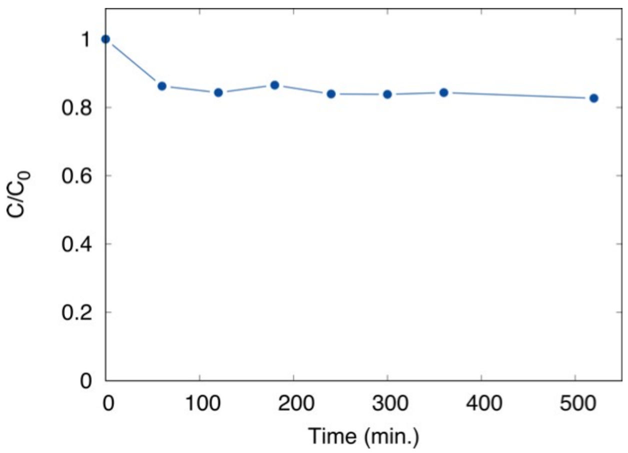

Figure S3 Normalized concentration of Orange II solution C/C_0 as a function of illumination time (no catalyst sample submerged in the solution).
